# Typhus Group Rickettsiosis, Germany, 2010–2017^1^

**DOI:** 10.3201/eid2407.180093

**Published:** 2018-07

**Authors:** Jessica Rauch, Philip Eisermann, Bernd Noack, Ute Mehlhoop, Birgit Muntau, Johannes Schäfer, Dennis Tappe

**Affiliations:** Bernhard Nocht Institute for Tropical Medicine, Hamburg, Germany (J. Rauch, P. Eisermann, B. Noack, U. Mehlhoop, B. Muntau, D. Tappe);; Tropenklinik Paul-Lechler-Krankenhaus, Tübingen, Germany (J. Schäfer)

**Keywords:** Rickettsia typhi, Rickettsia prowazekii, murine typhus, epidemic typhus, travel, PCR, serology, cytokines, vector-borne infections, zoonoses, bacteria

## Abstract

Typhus group rickettsiosis is caused by the vectorborne bacteria *Rickettsia typhi* and *R. prowazekii*. *R. typhi*, which causes murine typhus, the less severe endemic form of typhus, is transmitted by fleas; *R. prowazekii*, which causes the severe epidemic form of typhus, is transmitted by body lice. To examine the immunology of human infection with typhus group rickettsiae, we retrospectively reviewed clinical signs and symptoms, laboratory changes, and travel destinations of 28 patients who had typhus group rickettsiosis diagnosed by the German Reference Center for Tropical Pathogens, Hamburg, Germany, during 2010–2017. Immunofluorescence assays of follow-up serum samples indicated simultaneous seroconversion of IgM, IgA, and IgG or concurrence in the first serum sample. Cytokine levels peaked during the second week of infection, coinciding with organ dysfunction and seroconversion. For 3 patients, *R. typhi* was detected by species-specific nested quantitative PCR. For all 28 patients, *R. typhi* was the most likely causative pathogen.

Typhus group rickettsiosis (TGR) is caused by *Rickettsia typhi* and *R. prowazekii*, 2 Biosafety Level 3 organisms of the family *Rickettsiaceae*, which comprises obligate intracellular gram-negative zoonotic bacteria. *R. typhi* is responsible for murine typhus, the endemic fleaborne form of typhus, which is emerging in predominantly tropical coastal regions. *R. prowazekii* is responsible for epidemic louseborne typhus in temperate and tropical regions. *R. prowazekii* is classified as a Centers for Disease Control and Prevention category B bioweapon pathogen. 

The clinical picture of TGR caused by either pathogen is similar: fever, headache, and exanthema (*1*,*2*). Inoculation eschars, which are classically seen in patients with spotted fever group rickettsioses (SFGR), are usually absent in patients with TGR. Cardiac, pulmonary, and central nervous system (CNS) complications can occur during the course of infection (*1*,*2*). *R. typhi* infection is usually milder than *R. prowazekii* infection. Fatality rates among patients with untreated typhus are ≈4% (*3*,*4*) among patients with *R. typhi* infection and 13%–30% (*2*) among those with *R. prowazekii* infection.

To learn more about the immunology of human infection with typhus group rickettsiae, we retrospectively analyzed TGR cases diagnosed at the National Reference Center for Tropical Pathogens in Hamburg, Germany, during 2010–2017. We collected clinical data and analyzed patient serum. Antibody kinetics were determined from follow-up serum samples. Serum cytokine responses were measured by flow cytometry from all available serum samples. In addition, we used novel nested quantitative PCRs targeting the *prsA* genes of *R. typhi* and *R. prowazekii* on archived clinical material.

## Patients, Materials, and Methods

### Cases and Inclusion Criteria

We screened the database of the German Reference Center for Tropical Pathogens at the Bernhard Nocht Institute for Tropical Medicine in Hamburg for autochthonous and imported (travel- or migration-associated) TGR cases diagnosed from January 1, 2010, through December 31, 2017. Written general consent had been obtained from patients before the study. TGR cases were defined as a clinically compatible disease with >1 of the following laboratory test results: a positive PCR and sequencing result, seroconversion to TGR antigens in an indirect immunofluorescence assay (IFA), parallel TGR IgM and IgG detection in a single sample by IFA, or a single IFA IgG or total Ig titer of >320. In addition, antibodies against SFGR antigens, when available, had to be lower than TGR antigens in the IFA. Serologic testing results for leptospirosis (in-house ELISA), scrub typhus (in-house IFA), and dengue fever (in-house IFA) had to be negative.

### Serologic Assays

We performed in-house TGR IFA by using *R. typhi* strain Wilmington and *R. prowazekii* strain Madrid E grown in L929 mouse fibroblast cell culture. IFA reference values for *R. typhi* and *R. prowazekii* were <40 (IgM), <20 (IgA), and <80 (IgG and total Ig). In parallel, we performed in-house SFGR IFA with *R. conorii* strain 7 (ATCC VR-613) by using the same culture conditions and with reference values of <20 (IgM and IgA) and <40 (IgG and total Ig). All reference values were determined with serum from 200 healthy Caucasian blood donors.

### Molecular Assays

We performed panrickettsial real-time quantitative PCR (qPCR) targeting the *ompB* gene (*5*). A nested qPCR detecting specifically the *prsA* gene of *R. typhi* (*6*) was used, and a nested qPCR for amplifying the *prsA* gene of *R. prowazekii* was developed by using outer primers GCTTGCAGAAGAATTCTCTCTTG (forward) and GGCACAGGTTTTTTTTCAAGCAC (reverse) and nested primers CAGCGTCAAATGGTGGGATT (forward) and TGCCAACCGAAACTTGTTTTG (reverse) with established cycling conditions (*6*). Probes were 6FAM-ATCAATCAGGGCAATTAGTACCAGAA-BHQ1 for *R. typhi* and 6FAM-ATCAACCAGGGCAGTTAGTACCAGAA-BHQ1 for *R. prowazekii*. We used conventional gel PCRs for later sequencing, followed by BLAST analysis (http://www.blast.ncbi.nlm.nih.gov). We performed PCRs from DNA extracts from blood in EDTA, from the first archived serum sample if available, and in 1 case from a formalin-fixed paraffin-embedded liver biopsy sample.

### Cytokine Measurements

For all available serum samples, we analyzed serum cytokine responses by using LEGENDplex (BioLegend, Fell, Germany). For controls, we used 16 serum samples from healthy blood donors. For cytokine analysis, we assigned blood sampling dates from the patients as acute phase of infection (days 1–7 and days 8–14 of illness), prolonged phase (days 15–28 of illness), and convalescent phase (days 29–56 of illness).

## Results

**Figure 1:**
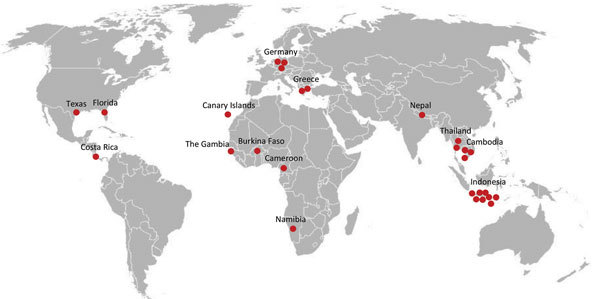
Countries and US states in which 27 of 28 patients acquired typhus group rickettsiosis diagnosed in Germany, 2010–2017. For 1 of the 28 patients, no information was available. Most infections were acquired in Southeast Asia, although 3 autochthonous cases were found in Germany. Each dot symbolizes 1 patient.

We identified 28 TGR patients (Table 1); age range was 4–80 years (mean age 38.3 years), and male:female ratio was 1.5:1. For 1 patient, no information about travel destination or medical history was available; only age and sex information was available. TGR infections had been acquired during travel (Figure 1), primarily to Southeast Asia (Indonesia, Thailand, Cambodia, [13 (46%) cases]); most infections were acquired in Indonesia (8 [29%] cases). Other cases were acquired in Europe (Germany, Greece, Canary Islands; 6 [21%] cases), Africa (The Gambia, Burkina Faso, Cameroon, Namibia; 4 [14%] cases), North America (Florida and Texas, USA; 2 [7%] cases), Costa Rica (1 case), and Nepal (1 case). Three infections were locally acquired in Germany by patients without a travel history (patients 9, 15, and 21). No patients had been asked about exposure to rats.

**Table 1:** Characteristics of 28 patients with typhus group rickettsiosis, Germany, 2010–2017*

Patient no.	Age, y/sex	Year of diagnosis	Travel history	PCR result†	Signs and symptoms	Hospitalized
1	26/M	2016	Indonesia (Java), Singapore	Negative	Fever, exanthema, femoral lymphadenitis	Yes
2	4/M	2016	The Gambia	Negative	Fever, myalgia, pneumonia	Yes
3	27/M	2015	Indonesia (Java, Bali, Gili, Lombok)	NA	Fever, headache	Yes
4	27/M	2015	Indonesia (Lombok, Komodo, Flores, Gili, Bali, Java)	NA	Fever, headache, exanthema	Yes
5	76/F	2015	Canary Islands	NA	Fever, exanthema, dysuria	Yes
6	21/F	2015	Thailand	NA	Fever, exanthema, genital herpes	No
7	27/F	2015	Cameroon	Negative	Fever, headache, acute kidney injury	Yes
8	26/F	2015	NA	NA	NA	NA
9	80/F	2015	No (Germany)	NA	Fever, headache, arthralgia, myalgia	No
10	42/M	2014	Thailand	Negative	Fever, exanthema	Yes
11	25/F	2014	Florida	NA	Cough, reddened ear	No
12	31/F	2014	Indonesia (Bali)	Negative	Fever, headache, exanthema	No
13	28/M	2014	Greece	*R. typhi*‡	Fever, fatigue	Yes
14	31/M	2014	Indonesia (Bali)	NA	Fever, exanthema	Yes
15	72/M	2014	No (Germany)	NA	Pneumonia, pleural effusion	Yes
16	34/F	2014	Costa Rica	NA	Headache	No
17	57/M	2013	Cambodia	*R. typhi*§	Fever, headache, exanthema, splenomegaly	Yes
18	75/M	2013	Texas	Negative	Exanthema, confusion, hallucination, fatigue	Yes
19	58/M	2013	Greece	NA	Fever, exanthema, arthralgia, fatigue	Yes
20	28/F	2013	Indonesia (Bali, Gili)	Negative	Fever, headache, myalgia, splenomegaly, fatigue	Yes
21	50/F	2012	No (Germany)	NA	Arthralgia	No
22	23/M	2012	Burkina Faso	Negative	Fever, diarrhea	Yes
23	21/M	2012	Namibia	NA	Fever, exanthema	No
24	53/M	2012	Cambodia	Negative	Fever, headache, exanthema, arthralgia	No
25	26/M	2011	Cambodia	NA	Fever, headache, exanthema	Yes
26	47/M	2011	Indonesia (Java, Bali)	Negative	Fever, headache, arthralgia, splenomegaly	No
27	22/M	2011	Indonesia (Bali, Lombok)	Negative	Fever, headache, arthralgia	Yes
28	34/F	2010	Nepal	*R. typhi*§	Fever, headache, exanthema, pneumonia	Yes

*NA, not available. †*Rickettsia typhi*–specific and *R. prowazekii*–specific nested *prsA* quantitative PCR. ‡Nested *R. typhi*–specific *prsA* quantitative PCR from liver biopsy sample, positive; from serum sample, negative. §Also positive *ompB* PCR from whole blood.

**Figure 2:**
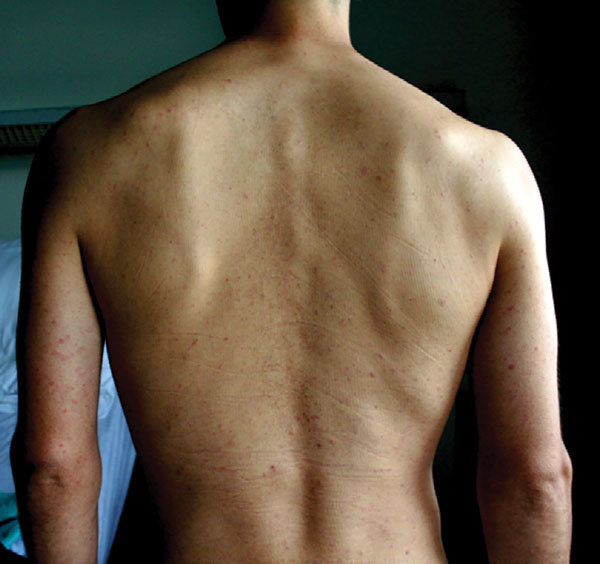
Typical exanthema in a typhus patient after travel to Thailand. The rash is maculopapular and nonpruritic.

Of note, patients were examined on different days of illness at different hospitals. At the time of initial examination, the most frequently reported sign or symptom was fever (79%), followed by exanthema (50%; Figure 2), headache (46%), myalgia/arthralgia (25%), cough/pneumonia (15%), and splenomegaly (11%). Only 6 (21%) patients had the classical TGR triad of fever, headache, and exanthema. The following were recorded in the medical records of 1 (4%) patient each: neurologic signs, lymphadenopathy, herpes simplex reactivation, acute kidney injury, dysuria, diarrhea, and ear redness. Hospitalization was necessary for 18 (64%) patients. Patients had received doxycycline (200 mg/d for 5–14 d) in the country of travel or after return; all recovered from infection without sequelae.

At the time of initial examination, laboratory changes were reported in the medical records of 23 patients and included increased levels of C-reactive protein (70%), liver enzymes (65%), lactate dehydrogenase (LDH, 13%), and creatine kinase (13%); thrombocytopenia (26%); anemia (17%); and leukocytosis (17%). A positive PCR for *R. typhi* was obtained for 3 (10.7%) patients: on days 7 and 10 of infection from whole blood by panrickettsial *ompB*-qPCR and *R. typhi*–specific nested *prsA*-qPCR (patients 28 and 17), and on day 10 from a liver biopsy sample by *R. typhi*–specific nested *prsA*-qPCR only (patient 13). We confirmed identity of *R. typhi* by sequencing of the 856 bp *ompB* gene fragment, which showed 100% identity to GenBank entries of *R. typhi* clone 4, strain B9991CWPP, strain TH1527, and strain Wilmington (GenBank accession nos. KF241858, CP003398, CP003397, and AE017197). Sequencing of the 140 bp *prsA* gene fragment showed 98% identity to the same strains. No positive PCR for *R. prowazekii* was obtained (Table 1).

The earliest that antibodies against TGR antigens were detected was day 7 of illness. The median day for seroconversion was day 12. The percentages of seroconversion and parallel and single antibody class detection against TGR antigens are shown in Table 2. For 5 patients, only limited serologic information was available because a limited quantity of serum had been stored for retesting. Antibody titers of any class varied among patients despite illness onset occurring on the same day. TGR-specific IgA titers were identical or lower and even undetectable for some patients but never higher than TGR-specific IgM titers except for 1 patient for whom specific IgA and IgG but not IgM were detected. No serologic differentiation between *R. typhi* and *R. prowazekii* was achieved by IFA; titer differences between the 2 species for all antibody classes were <2 for all patients.

**Table 2:** Serologic testing results for 28 patients with typhus group rickettsiosis, Germany, 2010–2017

Category	Result, % patients	Days of illness during which serologic results were obtained, range (median)
Observed seroconversion	18 (always with parallel IgM, IgA, and IgG detection)*	10–41 (12)†
Parallel IgM and IgG/total Ig detection in first sample	64	7–180 (10)‡
Parallel IgM, IgA, and IgG detection in first sample	46	7–39 (10)
Only 1 IgG or total Ig titer of >320 in any sample	14	10–99 (14)§

*1 patient showed 1 typhus group rickettsiae–specific IgM in the first sample, followed by IgA and IgG seroconversion in the follow-up sample. †1 patient returned 41 d after the initial visit and showed seroconversion. ‡For 1 patient, serum was collected 180 d after a disease compatible with typhus group rickettsiosis had occurred. §For 1 patient, serum was collected 99 d after a disease compatible with typhus group rickettsiosis had occurred.

**Figure 3:**
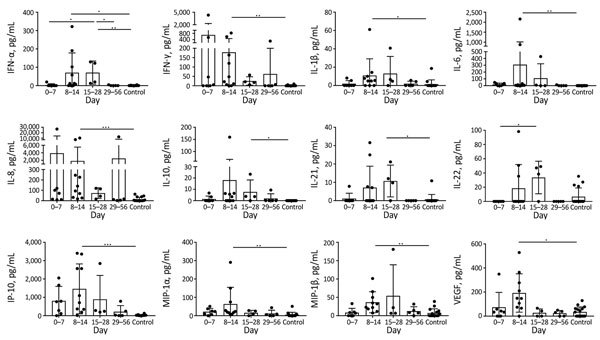
Cytokine and chemokine levels in serum from patients with imported and autochthonous typhus group rickettsiosis and controls, Germany, 2010–2017. Using a bead-based LEGENDplex assay (BioLegend, Fell, Germany), we analyzed 16 serum samples from healthy persons without rickettsial disease and 26 samples from 21 patients with typhus group rickettsiosis in parallel. We assigned 17 serum samples to the acute phase of illness (7 on days 0–7 and 10 on days 8–14), 4 to the prolonged phase, and 5 to the convalescent phase. Most serum cytokine levels started to increase in the first week of illness, peaked in the second week, and then started to decline again, except for IL-21 and IL-22, which reached their highest levels in the third week after symptom onset. Data are expressed as mean ± SD. Statistical analyses were performed by using the Kruskal-Wallis test and subsequently the Dunn multiple comparisons test. IFN, interferon; IL, interleukin; IP, interferon γ–induced protein; MIP, macrophage inflammatory protein; VEGF, vascular endothelial growth factor. Asterisks indicate statistically significant differences: *p<0.05; **p<0.01; ***p<0.001.

Serum cytokines (Figure 3) could be measured for 21 (75%) patients; for 11 patients, they were measured as kinetics at different time points (2–5 time points). Illness was determined to be in the acute phase according to 17 serum samples (7 on days 0–7 and 10 on days 8–14), in the prolonged phase for 4 samples, and in the convalescent phase for 5. Concentrations of interferon γ–induced protein (IP) 10 and vascular endothelial growth factor (VEGF) were elevated in the first week of the acute phase, peaked during the second week, and then declined. This trend was also observed for interleukin (IL) 8, except for patient 26, for whom IL-8 increased continuously (from 101 pg/mL on day 5 of illness and 245 pg/mL on day 12 up to 12,380 pg/mL on day 29). Serum levels of interferon-γ, IL-1β, IL-6, IL-8, IP-10, macrophage inflammatory proteins 1α and 1β, and VEGF were substantially increased over those in healthy controls in the second week of the acute phase of illness. The serum concentrations of IL-21 and IL-22 started to elevate during the second week of the acute phase, peaked during the prolonged phase, and were not detectable in the convalescent phase. Serum levels of interferon-α were substantially increased during the second and third weeks of illness; IL-10 was also elevated during this time. The serum concentrations of the following were comparable between controls and patients at all analyzed time points (data not shown): basic fibroblast growth factor, eotaxin, granulocyte colony stimulating factor, granulocyte-macrophage colony stimulating factor, IL-2, IL-4, IL-5, IL-9, IL-12p70, IL-13, IL-17A, IL-17F, monocyte chemotactic protein (MCP) 1, platelet-derived growth factor, RANTES (regulated on activation, normal T cell expressed and secreted), and tumor necrosis factor α. 

## Discussion

The rather benign course of illness and outcomes, the travel destinations, and the molecular identification of *R. typhi* for a few patients indicate that murine (endemic) typhus is the most likely diagnosis for all TGR patients in our study. We found no cases of definitive *R. prowazekii* infection (i.e., epidemic typhus). IFA testing did not allow for TGR species discrimination because no significant titer differences were noted between serum tested for *R. typhi* and *R. prowazekii*.

As expected, most TGR cases diagnosed in our study were travel associated. Nearly half of the infections were acquired in Southeast Asia. A surveillance study also found Southeast Asia to be the most common region of exposure for patients with murine typhus (*7*). Another study found Southeast Asia to rank second after Africa (*8*). We identified 3 autochthonous cases in patients from Germany, but the circumstances of infection and the exposure (rats or flea bites) could not be identified. For these 3 patients (50, 72, and 80 years of age), we could not exclude the possibility of a relapse of *R. prowazekii* infection (Brill-Zinsser disease) acquired during World War II or in early postwar Germany. For Europe, exposure to *R. typhi* had been previously recorded in Spain (*9*); Canary Islands and Greece (*8*); and Cyprus, Italy, France, Croatia and Slovenia (*10*).

The clinical signs of illness found in our study were undifferentiated, except for a maculopapular rash that had developed for half of the patients. The typical triad for murine typhus (fever, headache, and exanthema) occurred in less than one fourth of the patients reported here. According to similar findings by a study of comparable size in France, the triad was considered nonspecific (*8*); the triad occurred in one third of patients according to a study from Texas (*11*) and a recent review by others (*1*). In our study, nearly two thirds of the patients were hospitalized during travel or after return home. A similar high rate of 60% for TGR-associated hospitalization in Texas has been described (*11*). Complications, mostly pulmonary or CNS, have reached 26% among patients with murine typhus (*1*). In our study, the complication rate was 18%; complications included pneumonia, acute kidney injury, and CNS involvement.

In our study, laboratory data, which could not be retrieved in detail for all patients, often showed elevated levels of C-reactive protein, LDH, and liver enzymes, paralleled by thrombocytopenia. More than 70% of patients with murine typhus had increased liver enzyme and LDH levels, and nearly half had thrombocytopenia, as described in a recent review (*1*). Because many patients in our study had fever, exanthema, elevated liver enzymes, and thrombocytopenia, the differential diagnoses for travelers, especially to Asia, include scrub typhus, dengue fever, and leptospirosis. All patients in our study were negative for these infections. Thus, infection with *R. typhi* should be considered for patients with fever, headache, exanthema, and concurrent thrombocytopenia and elevated levels of liver enzymes, particularly patients who have recently traveled to Southeast Asia.

In our study, molecular detection of TGR species was positive for a liver biopsy and 2 whole blood samples but negative for archived serum. This finding is in line with the higher sensitivity of rickettsial PCR from whole blood or buffy coat than from serum (*12*).

Typically, diagnostic IgM and IgG are simultaneously detected 7–15 days after onset of symptoms (*13*) and titers are detected by IFA for 50% of patients at the end of the first week of symptoms and for nearly all after 2 weeks (*4*). In our study, the earliest detection of antibodies against TGR was day 7 of illness, and the median day of seroconversion was day 12. Seroconversion, which is delayed in patients with TGR rickettsiosis, involved IgM, IgA, and IgG simultaneously. In nearly half of the patients in our study, antibodies of these 3 classes were detected in parallel against TGR in the first serum sample collected. It remains tempting to speculate about an initial depression of B-cell responses by TGR rickettsiae. IgA testing had been included in the study to test for possible earlier seroconversion with antibodies in this class, but IgA detection proved less sensitive than IgM detection. Of note, because serum had not been collected from all patients on the same day and was not collected every day, the time points can only serve as estimates.

We report data on the systemic inflammatory response in patients with TGR during 8 weeks after illness onset. Data on circulating levels of inflammatory mediators are available for only a few SFGR diseases, probably because of the limited availability of patient samples. Most cytokine and chemokine elevations in TGR patients in our study occurred in the second week of infection, coinciding with organ dysfunction and seroconversion. The mechanisms expected to contribute to the vascular permeability observed in patients with clinical disease include the effects of inflammatory cells and their mediators (*14*). Of note, vascular dysfunction and damage in the infected host most likely contribute to the pathogenesis of human rickettsial diseases. Because endothelial cells are the major target cells for rickettsial infections (*15*) and have emerged as key immune-reactive cells involved in host defense and inflammation (*16*,*17*), prior studies have focused on the behavior and inflammatory phenotype of these cells after infection in vitro. Endothelial cells react to infection with SFGR species by increased expression of cytokines such as IL-1 and IL-6 and chemokines such as IL-8 and MCP-1, which favor the migration of leukocytes (*18*–*20*). Experiments with cultured endothelial cells showed that they also become activated by *R. prowazekii*, which induces the expression of proinflammatory cytokines and chemokines (*21*). Endothelial cells upregulate the expression of the cytokines tumor necrosis factor-α, IL-1α, and IL-6 and the chemokines IP-10, MCP-1, and RANTES, which leads to transmigration of peripheral blood mononuclear cells that acquire an inflammatory transcriptional profile. Infection of endothelial cells with *R. typhi* resulted in enhanced expression of IP-10, MCP-1, and RANTES in infected endothelial cells; the expression of IL-8 was also upregulated (*22*). Although different intracytoplasmic behavior of SFGR and TGR pathogens has been shown (*22*–*25*), our findings indicate that endothelial cells react in a comparable way after infection with pathogens of either group. In fact, the elevations of the chemokines IL-8, IP-10, macrophage inflammatory protein 1α, IL-6, and IL-10 that we found in the serum of the patients in our study during the acute phase of illness were also detectable in patients with SFGR, such as African tick bite fever caused by *R. africae* and Mediterranean spotted fever caused by *R. conorii* (*26*–*28*). Moreover, inflammatory cytokines such as IL-1β, IL-6, and IL-8 upregulate VEGF expression (*29*). Of note, the serum concentrations of all these mediators were substantially elevated during the same period in the patients in our study (precisely in the second week of illness). VEGF and IL-8 are important mediators of angiogenesis and might contribute to initiation of repair mechanisms after endothelial damage through rickettsial growth and spread (*30*). In addition, IL-8 induces neutrophil mobilization and activation (*31*). Neutrophils are the first cells of the innate immune system that migrate to the site of infection and participate in bacterial defense.

In our study, interferon-γ levels were substantially elevated in the serum of patients in the acute phase of disease and seem to play a crucial role in antirickettsial immune responses. Interferon-γ has protective features in host defense during infection of susceptible mouse strains with SFGR and TGR species (*32*–*37*), and it adversely affects the growth of TGR species in various host cells (*36*–*39*). Therefore, the early interferon-γ response could activate intracellular bactericidal mechanisms to further control the spread of infection.

The concentrations of IL-21 and IL-22 in the serum of TGR patients started to increase in the second week of illness, peaked in the third week, and were no longer detectable 1 month after symptom onset. IL-22 seems to have protective functions because it increases the production of neutrophilic granulocyte-attracting chemokines such as IL-8, protects tissues from damage, and enhances tissue regeneration (*40*,*41*). IL-22–responsive cells are distributed throughout the body in several organs including those from the digestive (pancreas, liver, colon) and respiratory (lung, trachea) systems and the skin (*42*). Whether endothelial cells express the IL-22 receptor complex remains to be determined. IL-22 is produced by several types of cells of the lymphoid lineage and include activated T-cells as well as innate lymphoid cells such as natural killer cells, lymphoid tissue inducer cells, and lymphoid tissue inducer–like cells (*43*–*50*). Which cell types are responsible for IL-22 secretion and the protective potential of this cytokine during rickettsial infections remains to be elucidated.

In conclusion, our data broaden the knowledge of TGR immunology and diagnosis and shed light on immunologic changes that occur during successive weeks of illness. However, more investigations of immunologic changes, including analyses of human B and T cells, are needed.
